# Transcriptomic analysis reveals hub genes and pathways in response to acetic acid stress in *Kluyveromyces marxianus* during high-temperature ethanol fermentation

**DOI:** 10.1007/s44154-023-00108-y

**Published:** 2023-07-26

**Authors:** Yumeng Li, Shiqi Hou, Ziwei Ren, Shaojie Fu, Sunhaoyu Wang, Mingpeng Chen, Yan Dang, Hongshen Li, Shizhong Li, Pengsong Li

**Affiliations:** 1 Beijing Key Lab for Source Control Technology of Water Pollution, College of Environmental Science and Engineering, Beijing Forestry University, Beijing, 100083 China; 2 Engineering Research Center for Water Pollution Source Control & Eco-Remediation, College of Environmental Science and Engineering, Beijing Forestry University, Beijing, 100083 China; 3 Institute of New Energy Technology, Tsinghua University, Beijing, 100084 China

**Keywords:** *Kluyveromyces marxianus*, Acetic acid, Transcriptomics, Protein–protein interaction network

## Abstract

The thermotolerant yeast *Kluyveromyces marxianus* is known for its potential in high-temperature ethanol fermentation, yet it suffers from excess acetic acid production at elevated temperatures, which hinders ethanol production. To better understand how the yeast responds to acetic acid stress during high-temperature ethanol fermentation, this study investigated its transcriptomic changes under this condition. RNA sequencing (RNA-seq) was used to identify differentially expressed genes (DEGs) and enriched gene ontology (GO) terms and pathways under acetic acid stress. The results showed that 611 genes were differentially expressed, and GO and pathway enrichment analysis revealed that acetic acid stress promoted protein catabolism but repressed protein synthesis during high-temperature fermentation. Protein–protein interaction (PPI) networks were also constructed based on the interactions between proteins coded by the DEGs. Hub genes and key modules in the PPI networks were identified, providing insight into the mechanisms of this yeast's response to acetic acid stress. The findings suggest that the decrease in ethanol production is caused by the imbalance between protein catabolism and protein synthesis. Overall, this study provides valuable insights into the mechanisms of *K. marxianus*'s response to acetic acid stress and highlights the importance of maintaining a proper balance between protein catabolism and protein synthesis for high-temperature ethanol fermentation.

**Supplementary Information:**

The online version contains supplementary material available at 10.1007/s44154-023-00108-y.

## Introduction

The use of biofuels has become increasingly important in recent years, with bioethanol being one of the most widely used alternatives to fossil fuels. Bioethanol has the potential to reduce emissions of air pollutants (Goldemberg 2007; Salvo et al. 2017) and CO_2_ (Scully et al. 2021), making it a promising alternative to fossil fuels. However, the use of sucrose- and starch-rich crops as feedstocks for bioethanol production conflicts with food and feed production. To overcome this issue, cellulosic ethanol has been developed as a 2nd generation bioethanol, which utilizes lignocellulose from forestry and agricultural residues as feedstocks.

The production of cellulosic ethanol involves pretreatment, saccharification and fermentation. While *Saccharomyces cerevisiae* has been widely used for industrial ethanol fermentation, its inability to utilize pentose limits its application in cellulosic ethanol production. In contrast, the thermotolerant yeast *Kluyveromyces marxianus* has several advantages, including thermotolerance, high growth rate and a broad substrate spectrum. Its ability to ferment both hexose and pentose without genetic modification makes it a suitable candidate for cellulosic ethanol production (Fonseca et al. 2008; Nonklang et al. 2008). Moreover, *K. marxianus* can be employed for high-temperature fermentation, which reduces cooling costs, minimizes the risk of contamination, and allows for more efficient simultaneous saccharification and fermentation (Limtong et al. 2007). Overall, *K. marxianus* has great potential for use in cellulosic ethanol production, and its unique characteristics make it a promising alternative to *S. cerevisiae* in the biofuel industry.

Fermentation inhibitors such as weak acids, furan aldehydes and phenolic compounds generated during pretreatment of lignocellulosic feedstocks can hinder microbial growth, metabolism and ethanol production (Wang et al. 2018). Among these inhibitors, acetic acid is a major fermentation inhibitor produced during acid-catalyzed hydrolysis of lignocellulose (An et al. 2015). Acetic acid has been found to affect the growth and metabolism of *K. marxianus* (Martynova et al. 2016; Rugthaworn et al. 2014), and our previous studies have shown that *K. marxianus* produces more acetic acid during high-temperature fermentation than at lower temperatures, leading to incomplete glucose consumption and inhibited ethanol fermentation (Fu et al. 2019; Li et al. 2021). Despite the critical role of acetic acid in limiting high-temperature ethanol fermentation of *K. marxianus*, the mechanisms underlying *K. marxianus*' response to acetic acid during high-temperature fermentation have not been fully elucidated. Therefore, studying the transcriptomic responses of *K. marxianus* to acetic acid during high-temperature ethanol fermentation is necessary to understand its response mechanism and identify potential targets for improving ethanol production in *K. marxianus.*

The development of high-throughput sequencing technology has enabled in-depth exploration of the response mechanisms of yeasts to acetic acid stress. However, most studies have focused on *S. cerevisiae*, leaving significant research gaps in the response and tolerance mechanism of *K. marxianus* to acetic acid stress. Therefore, revealing the response mechanism of *K. marxianus* to acetic acid stress is crucial to improve its tolerance and promote its application in high-temperature ethanol fermentation.

In this study, we analyzed the transcriptome changes of *K. marxianus* under acetic acid stress, revealing its response mechanisms to this stress. The findings of this study provide a scientific basis for the construction of acetic acid-tolerant *K. marxianus* strains, which can further enhance its application in ethanol fermentation.

## Results

### Acetic acid repressed high-temperature ethanol fermentation of *K. marxianus*

**Fig. 1:**
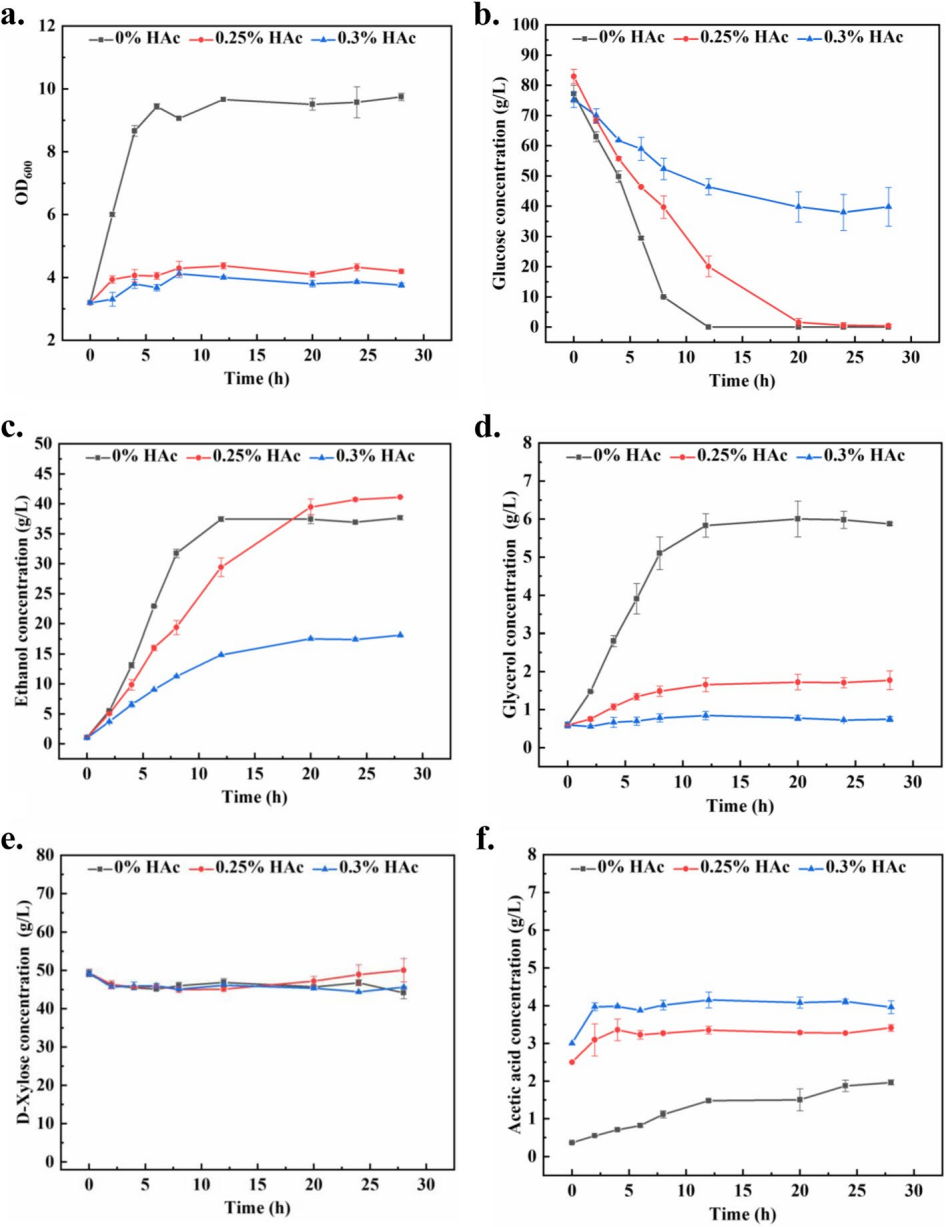
Changes in (**a**) OD_600_, concentrations of (**b**) glucose, (**c**) ethanol, (**d**) glycerol, (**e**) xylose and (**f**) acetic acid during fermentation at 45°C under different concentrations of acetic acid

The cell concentration was monitored throughout the high-temperature ethanol fermentation process. When no acetic acid was added, the OD_600_ reached a peak of ~ 9.4 within 8 h (Fig. 1a). When acetic acid was added to the fermentation media, however, the growth of *K. marxianus* was significantly repressed, with maximum OD_600_ of ~ 4.3 and ~ 4.0 appeared at 8 h in the groups treated with 0.25% and 0.3% acetic acid, respectively (Fig. 1a).

According to the high-performance liquid chromatography (HPLC) results, the metabolism of *K. marxianus* was also inhibited by acetic acid. Glucose consumption in the control group was the fastest, with the glucose consumed completely within 10 h, while the glucose in the group treated with 0.25% acetic acid was completely consumed within more than 20 h (Fig. 1b). Glucose consumption in the group treated with 0.3% acetic acid was not only the slowest, but it also stopped after 20 h, with around 40 g/L glucose remained unconsumed (Fig. 1b). Acetic acid treatment also slowed down ethanol generation. The ethanol concentration in the control group reached a peak of 37 g/L within 12 h, while the ethanol concentrations in the groups treated with 0.25% and 0.3% acetic acid reached corresponding maximum values of 40 and 18 g/L at around 24 h, respectively (Fig. 1c). Interestingly, total glycerol production of the group treated by 0.25% acetic acid was less than that of the control group (Fig. 1d), making the conversion yield of the former (0.49 g ethanol/g sugar) higher than that of the latter (0.45 g ethanol/g sugar) (Table 1). To our surprise, although treatment with 0.25% acetic acid inhibited cell growth and slowed down ethanol generation, the specific productivity in this group was significantly higher than the that in the control group (Table 1). The growth rates and metabolite production rates are in the Fig. S1, the consumption rate of xylose was almost zero, and within 6 h, the yeast's growth rate and metabolite production rate reached their peaks.

**Table 1:** Fermentation results (0–24 h) in this study

Fermentation parameters	0% HAc	0.25% HAc	0.3% HAc
Initial OD_600_	3.19	3.19	3.19
Final OD_600_	9.74	4.19	3.76
Volumetric productivity (g/L h^−1^)	1.31	1.43	0.61
Specific productivity (g/OD_600_ h^−1^)	0.13	0.34	0.16
Conversion yield (g ethanol/g sugar)	0.45	0.49	0.44

### Descriptive statistics of RNA-seq data

During the early stage of acetic acid stress in yeast cells, genome-wide alterations in transcription occur (Geng et al. 2017). In order to reveal the transcriptomic responses of *K. marxianus* induced by acetic acid, the cells in the group treated with 0.25% acetic acid and the control group were sampled at 2 h, and then subjected to total RNA extraction. Both 0.25% and 0.3% acetic acid could significantly inhibit cell growth (Fig. 1a), but the concentrations of produced ethanol were almost the same between 0.25% acetic acid treated group and the control group (Fig. 1c). Therefore, to rule out the differences in gene expression caused by different concentrations of ethanol, the concentration of 0.25% acetic acid was chosen for RNA-seq. Based on RNA quality evaluation (Table S1), the RNA samples were qualified for library construction. Paired-end sequencing generated 297.7 million raw reads, ranging from 42.3 to 56.9 million per sample. After filtering, 41.7–56.3 million clean reads were obtained per sample (Table S2). HISAT2 alignment showed 92.97–95.07% of clean reads uniquely mapped to the reference genome (Table S3). We also performed principal component analysis (PCA) to investigate if samples with the same treatment cluster together. According to the PCA result, the first two principal components explained more than 89% of the variability among the samples, and acetic acid treated samples and control samples were grouped in different clusters (Fig. S2). This result indicated that the transcriptome profiles was significantly changed after acetic acid treatment. The acetic acid treated samples fell in the negative direction of the PC1 axis, while the control samples fell in the positive direction. In PC2, one sample in the control group did not cluster with others.

### Identification of DEGs and functional enrichment analysis

**Fig. 2:**
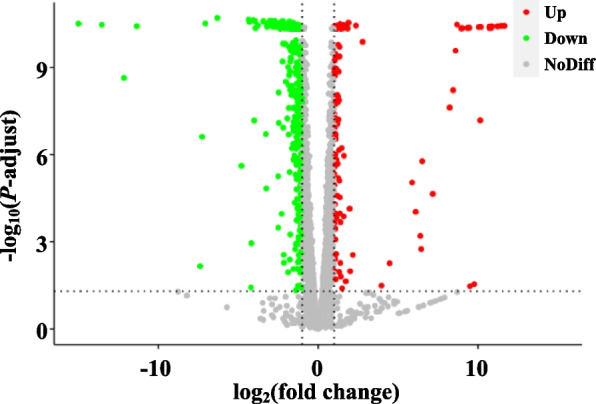
Volcano plot of the differentially expressed genes (DEGs) induced by acetic acid

According to differential expression analysis, 611 DEGs (fold change > 2 or < 0.5, *P*-adjust < 0.05) were identified in the samples treated with 0.25% acetic acid compared with the control group, with 166 up-regulated and 445 down-regulated (Fig. 2a). Among the up-regulated DEGs, those with fold changes > 10 accounted for 18.07%, those with fold changes between 5 and 10 accounted for 35.54%, and those with fold changes between 2 and 5 accounted for 46.39%. Among the down-regulated DEGs, those with fold changes < 0.1 accounted for 4.49%, those with fold changes between 0.1 and 0.2 accounted for 8.31%, and those with fold changes between 0.2 and 0.5 accounted for 87.19%. The DEGs were functionally categorized into GO functional classes and KEGG pathways (Fig. S3).

**Fig. 3:**
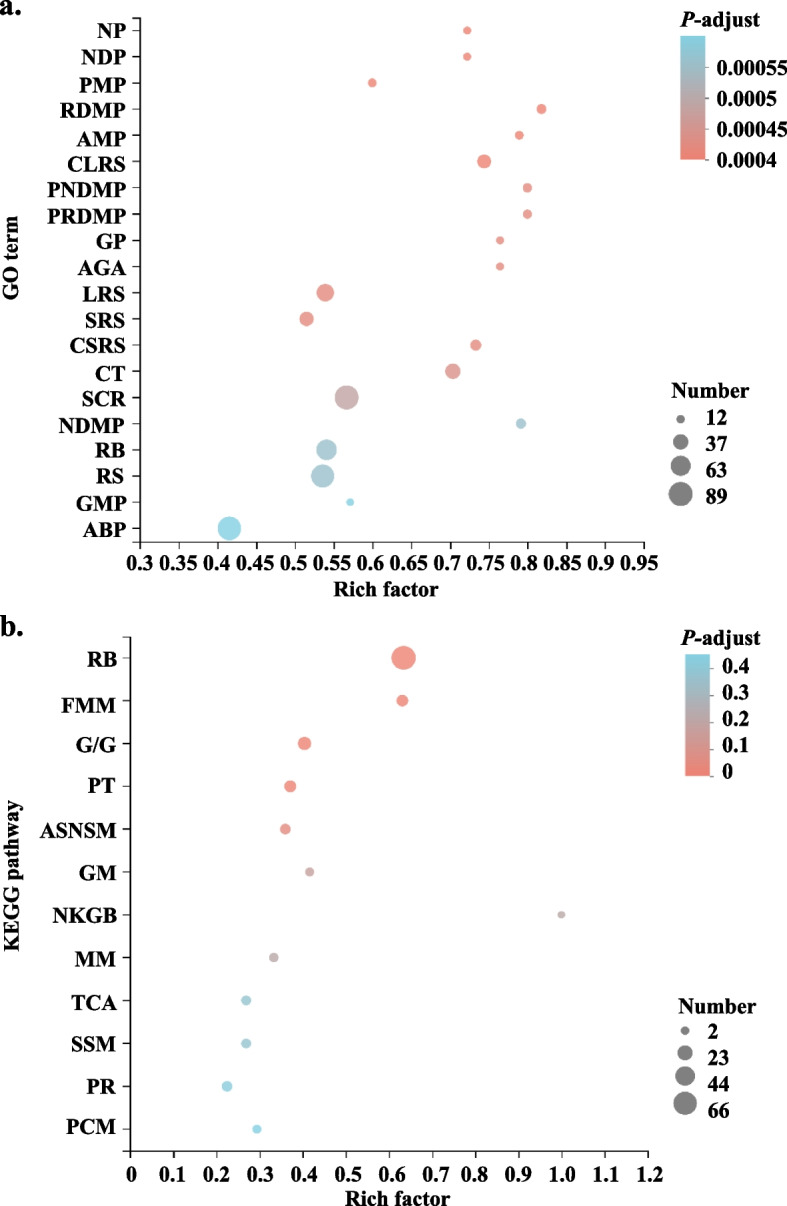
Enrichment analysis of the DEGs in this study. **a** GO terms enriched in the DEGs; **b** KEGG pathways enriched in the DEGs. Rich factor is the ratio of DEG number annotated in this GO term (or KEGG pathway) to all gene number annotated in this GO term (or KEGG pathway). Greater rich factor means greater effect of acetic acid on the analyzed GO term (or KEGG pathway). NP: nucleotide phosphorylation; NDP: nucleoside diphosphate phosphorylation; PMP: pyruvate metabolic process; RDMP: ribonucleoside diphosphate metabolic process; AMP: ADP metabolic process; CLRS: cytosolic large ribosomal subunit; PNDMP: purine nucleoside diphosphate metabolic process; PRDMP: purine ribonucleoside diphosphate metabolic process; GP: glycolytic process; AGA: ATP generation from ADP; LRS: large ribosomal subunit; SRS: small ribosomal subunit; CSRS: cytosolic small ribosomal subunit; CT: cytoplasmic translation; SCR: structural constituent of ribosome; NDMP: nucleoside diphosphate metabolic process; RB: ribosome; RS: ribosomal subunit; GMP: glucose metabolic process; ABP: amide biosynthetic process; FMM: fructose and mannose metabolism; G/G: Glycolysis/Gluconeogenesis; PT: proteasome; ASNSM: amino sugar and nucleotide sugar metabolism; GM: galactose metabolism; NKGB: neomycin, kanamycin and gentamicin biosynthesis; MM: methane metabolism; TCA: citrate cycle (TCA cycle); SSM: starch and sucrose metabolism; PR: peroxisome; PCM: porphyrin and chlorophyll metabolism

To further analyze the functions of the DEGs induced by acetic acid, GO and KEGG enrichment analyses were conducted. According to the result of GO enrichment analysis (Fig. 3a and Table S4), the significantly enriched GO terms in the DEGs were related to energy metabolism, such as pyruvate metabolic process (GO:0006090), glycolytic process (GO:0006096), glucose metabolic process (GO:0006006), and ATP generation from ADP (GO:0006757), which are closely linked to energy metabolism. GO terms related to protein synthesis, such as small ribosomal subunit (GO:0015935), large ribosomal subunit (GO:0015934) and cytosolic small ribosomal subunit (GO:0022627), suggest the involvement of ribosome in the cellular response to acetic acid. Furthermore, the result of KEGG pathway enrichment show that ribosome (map03010), fructose and mannose metabolism (map00051), glycolysis/gluconeogenesis (map00010), TCA cycle (map00020), proteasome (map03050), etc. were enriched in the DEGs (Fig. 3b and Table S5).

Therefore, based on the enrichment results, it can be inferred that acetic acid may act on mitochondria, ribosome-related molecular functions, cellular components, biological processes, etc. to influence the energy conversion process in cells and thus the growth and metabolism of microorganisms.

### PPI network analysis

**Fig. 4:**
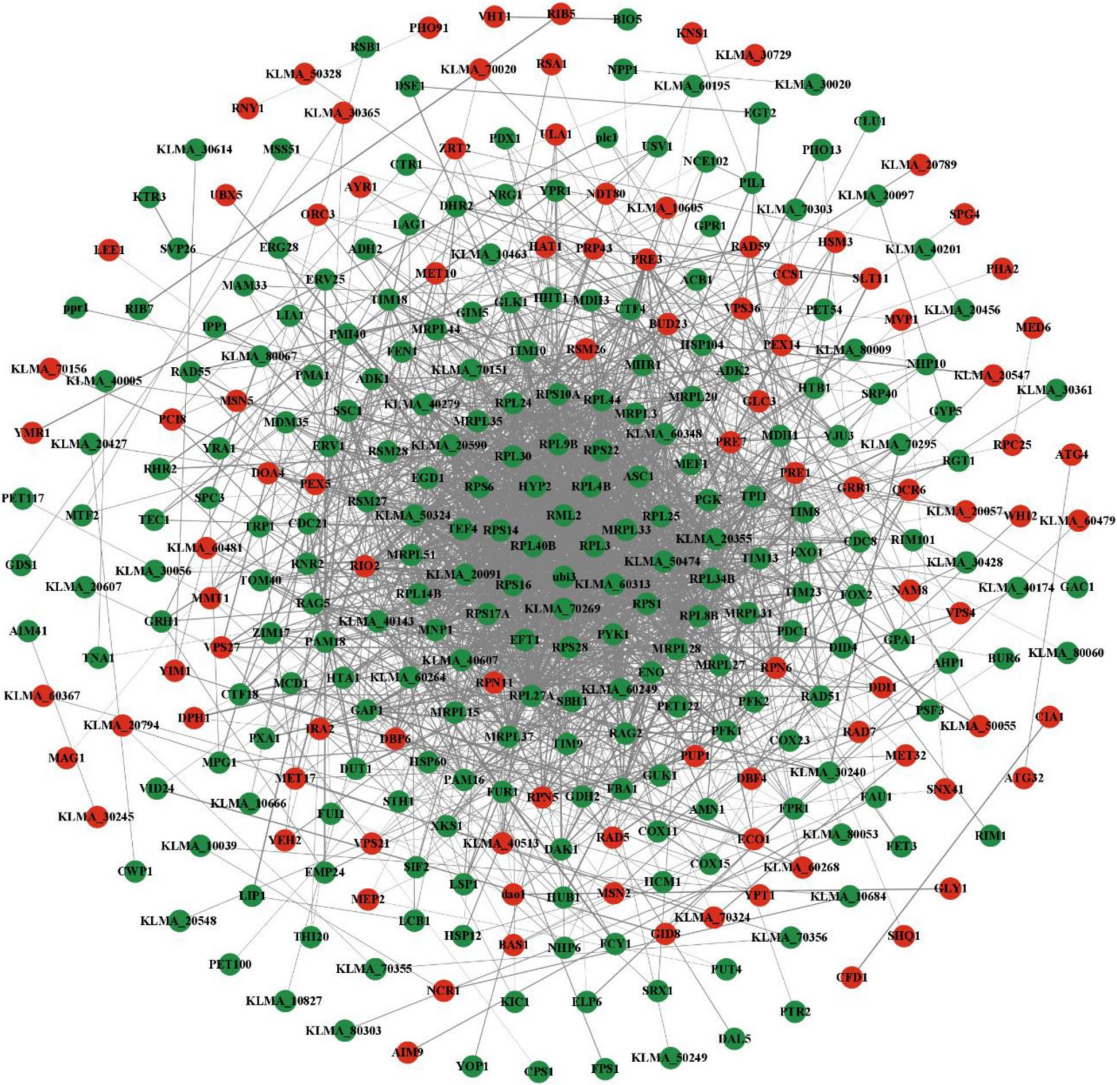
Protein–protein interaction (PPI) network of the proteins coded by the DEGs in this study. Red nodes represent proteins coded by up-regulated DEGs; green nodes represent proteins coded by down-regulated DEGs; edges represent protein–protein interactions, and thicker edges indicate stronger interactions

In order to further elucidate the response mechanism to acetic acid stress, PPI networks of all DEGs, up-regulated DEGs and down-regulated DEGs were constructed, respectively. Four hundred and seventeen proteins associated with the DEGs were matched with the STRING database and used to construct the PPI networks. The threshold of interaction score was set to > 0.4 and the unconnected nodes were hidden. The constructed PPI networks of all DEGs consisted of 329 nodes and 2041 edges (Fig. 4), while those of up-regulated DEGs and down-regulated DEGs consisted of 73 nodes, 116 edges (Fig. S4), and 225 nodes, 1612 edges (Fig. S5), respectively. In the PPI network of the proteins coded by all the DEGs, there were many tightly interconnected nodes corresponding to down-regulated genes associated with ribosome (such as *RPL3*, *PRL4B*, *RPL25*, *RPS6*, *RPL40B* and *RPS1*), while the nodes corresponding to up-regulated genes were relatively loosely connected (Fig. 4). Based on the PPI network of up-regulated DEGs, the nodes corresponding to proteasome-related genes (such as *RPN5*, *RPN6* and *RPN11*) exhibited tight interconnections (Fig. S4), indicating that these genes were coordinately involved in the cellular response to acetic acid stress.

**Fig. 5:**
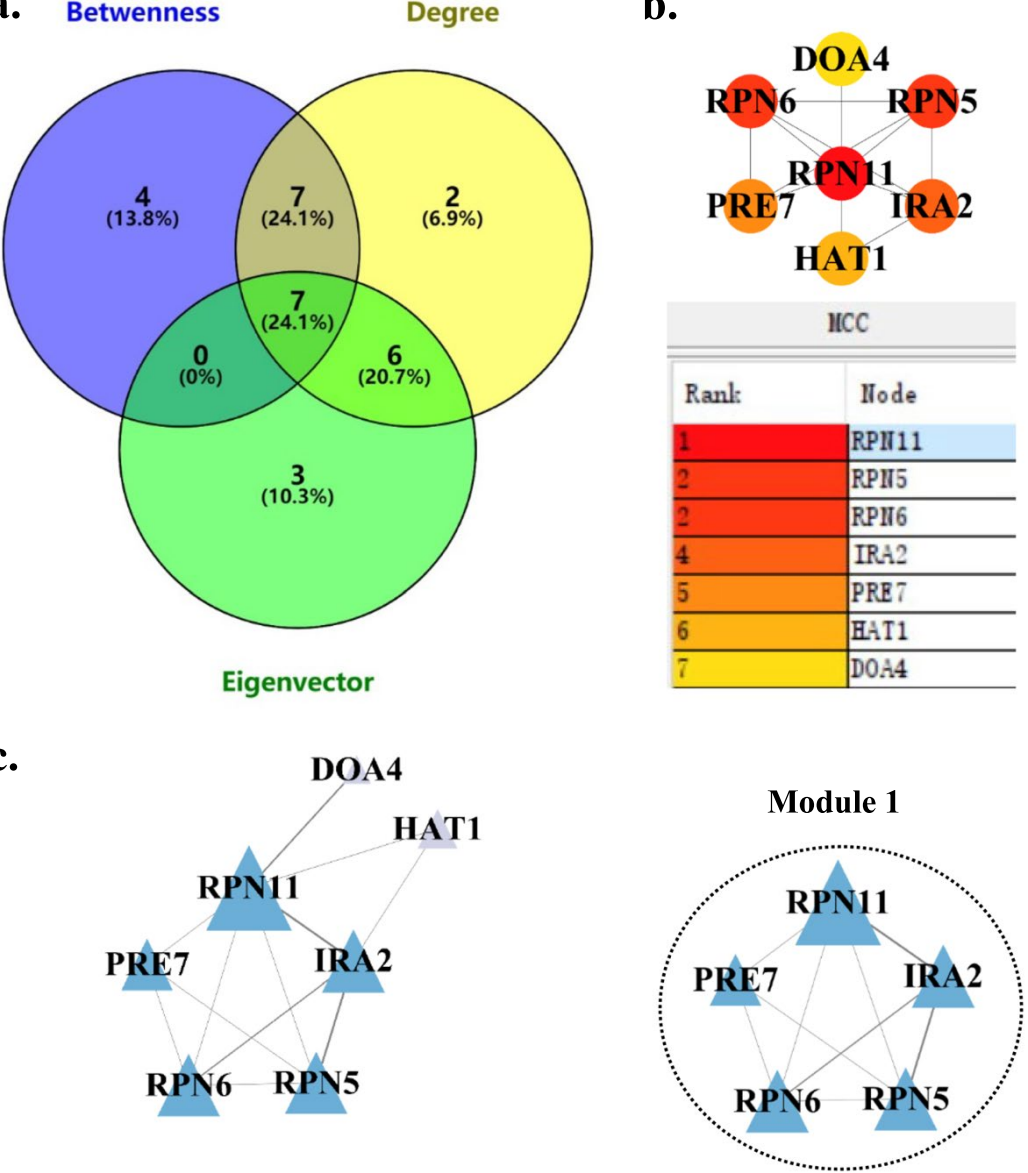
Major PPI networks of up-regulated DEGs and module analysis. **a** Venn diagram showing nodes with values of centrality parameters (degree, betweenness, and eigenvector) above average. **b** Major PPI network of up-regulated hub genes and MCC ranking of these genes. Node color reflects the degree of connectivity, the redder a node, the greater its MCC value. **c** MCODE analysis. Module 1: score = 4.5

**Fig. 6:**
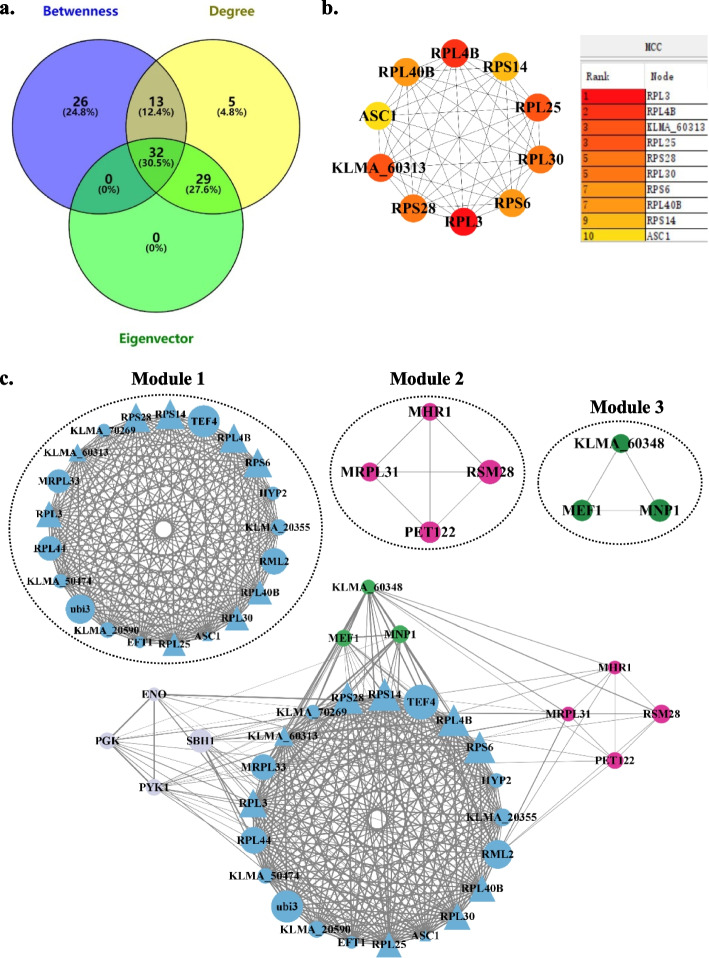
Major PPI networks of down-regulated DEGs and module analysis. **a** Venn diagram showing nodes with values of centrality parameters (degree, betweenness, and eigenvector) above average. **b** Major PPI network of top 10 down-regulated hub genes with highest MCC values and MCC ranking of these genes. Node color reflects the degree of connectivity, the redder a node, the greater its MCC value. **c** MCODE analysis. Module 1: score = 20.8; Module 2: score = 4; Module 3: score = 3. Triangles represent hub genes; the sizes of the circles indicate the MCC values

To obtain the major PPI network of up-regulated DEGs, the topological connectivity of each node was determined based on the centrality parameters degree, betweenness and eigenvector. According to the analyses of centrality parameters, twenty-two, eighteen and sixteen nodes were identified from the PPI network of up-regulated DEGs with the values of degree, betweenness and eigenvector above average, respectively. Seven nodes showed all these three centrality parameters above average and formed the major PPI network of up-regulated DEGs with 12 edges, which was equivalent to 24.1% of the PPI network of up-regulated DEGs (Fig. 5a and Fig. S6). These hub genes were ranked by MCC value (Fig. 5b and Table S6). A single module was identified from this major PPI network (Fig. 5c). Similarly, the major PPI network of down-regulated DEGs with 32 nodes and 316 edges was obtained (Fig. 6a and Fig. S7). The top 10 hub genes with higher MCC values were identified and sequentially ordered (Fig. 6b and Table S7). In addition, three significant modules were identified via the MCODE plugin (Fig. 6c).

To further investigate the functions of genes in the identified modules, we performed GO and KEGG enrichment analyses for these genes. We found that GO terms and KEGG pathways related to proteasome were enriched in the only identified module of major PPI network of up-regulated DEGs (Tables S8-S9). For the major PPI network of down-regulated DEGs, GO terms associated with ribosome, mitochondrial ribosome and mitochondrial translation were enriched in modules 1, 2 and 3, respectively, and a KEGG pathway related to ribosome was also enriched in module 1, but no KEGG pathway was enriched in modules 2 and 3 due to the limited numbers of nodes in these modules. These results indicated that acetic acid stress promoted protein catabolism but repressed protein synthesis, which affected the growth and metabolism of *K. marxianus* and led to the decrease of ethanol production. In order to further verify this inference, we extracted and quantitatively determined total protein content in the yeast cells expose to 0.25% acetic acid (treatment group) and the control group. Compared with the control group, the protein content in *K. marxianus* decreased by 55.41% under acetic acid stress (Fig. S8), which confirmed the bioinformatic results.

## Discussion

*K. marxianus*, a thermotolerant yeast capable of thriving at 45℃, exhibits potential for industrial ethanol production, in contrast to other yeasts, such as *K. lactis* and *S. cerevisiae* (Kosaka et al. 2022). Despite its advantageous features, acetic acid, a byproduct of acid-catalyzed hydrolysis of lignocellulose, constitutes one of the main fermentation inhibitors affecting growth, metabolism and ethanol production in *K. marxianus*, especially under high-temperature fermentation conditions. Undissociated acetic acid permeates through the plasma membrane and splits into H^+^ and CH_3_COO^−^, thereby acidifying the cell cytoplasm and impeding cellular metabolic activities, eventually leading to cell death (Arneborg et al. 2000; Casal et al. 1996). Although previous studies in *S. cerevisiae* exposed to acetic acid have documented significant changes in gene expression at the transcriptional level (Geng et al. 2017), little is known about the transcroptomic changes and the involved molecular mechanisms that *K. marxianus* utilizes to counteract acetic acid stress.

In the current study, we conducted high-temperature ethanol fermentation and performed PCA analysis to evaluate the effects of acetic acid treatment on the samples. The results indicate that the PC1 axis represents the primary source of variation between the acetic acid-treated samples and the control samples. The acetic acid treatment potentially exerts a significant influence on the transcriptome profile of the samples. We then analyzed the transcriptome of *K. marxianus* cells exposed to 0.25% acetic acid and identified 611 DEGs, with 166 up-regulated and 445 down-regulated DEGs. GO and KEGG enrichment analyses were performed on DEGs to understand their biological functions. GO terms related to ribosome were significantly enriched under acetic acid stress (such as GO:0022625, GO:0015934, GO:0015935, GO:0022627, GO:0005840, GO:0044391, GO:0002181 and GO:0003735) (Fig. 3a and Table S4). Meanwhile, KEGG pathway ribosome (map03010) was significantly enriched (Fig. 3b and Table S5). Ribosomes are highly involved in the synthesis of proteins, and thus it can be hypothesized that they may be a primary target of acetic acid treatment. A study conducted on *S. cerevisiae* reported that exposing the cells to acetic acid (150 mM, pH 3.0) resulted in reduced expression of numerous ribosomal 40S and 60S subunits, leading to a considerable decline in protein synthesis (Dong et al. 2017). Furthermore, the GO terms and KEGG pathways enriched were primarily related to energy metabolism (including GO:0006090, GO:0046031, GO:0006096, GO:0006757, map00010 and map00020). Since mitochondria are an important center for energy production, metabolism, signaling and cell cycle (McBride et al. 2006), they may potentially be another target for acetic acid treatment. This conclusion is supported by a study showing that exposure of *S. cerevisiae* cells to acetic acid (300 mM) causes down-regulation of genes encoding mitochondrial ribosomal proteins at the transcriptional level, indicating that mitochondria play a vital role in the cellular response to acetic acid (Li and Yuan 2010). Additionally, proteasome (map03050) and peroxisome (map04146) could potentially be further targets in response to acetic acid treatment (Fig. 3b and Table S5). These findings indicate that acetic acid may impact various aspects of cellular metabolism.

We further constructed PPI networks of all identified DEGs (Fig. 4 and Figs. S4-S5) and identified hub nodes in major PPI networks of up-regulated (Fig. S6) and down-regulated (Fig. S7) DEGs. This enabled us to infer potential regulatory genes and dominant pathways in response to acetic acid. The hub nodes of down-regulated DEGs are mainly relevant to cellular components, biological processes and molecular functions of ribosomes (Table S7), and all 10 hub nodes are in functional module 1 of the major PPI network of down-regulated DEGs (Fig. 6c), which are associated with ribosome-related GO terms and KEGG pathways (Table S8 and Table S9). A similar trend was observed in the transcriptomic analysis of formic acid stress response in *S. cerevisiae*, with the expression levels of genes involved in ribosome synthesis being down-regulated (Zeng et al. 2022). Ribosomal proteins play a crucial role in cell growth and proliferation (Petibon et al. 2021). Under acetic acid stress, the down-regulation of genes such as *RPL3*, *PRL4B*, *RPL25*, *RPS6*, *RPL40B* and *RPS1* may lead to incomplete ribosome structure, inhibiting the translation process, and lowering the levels of essential proteins. This ultimately affects the growth and metabolic activity of *K. marxianus* and reduces its ethanol production. The GO terms of module 2 and module 3 are related to mitochondrial ribosomes and mitochondrial translation (Table S8). Mitochondria, as an essential eukaryotic organelle, has its own genome that encodes proteins necessary for normal mitochondrial function. Some mitochondrial proteins can regulate mitochondrial acetate levels and play a significant role in acetate detoxification, which is critical for mitochondrial function (Fleck and Brock 2009; Orlandi et al. 2012). Mitochondrial ribosomes are responsible for protein synthesis inside mitochondria. The genes *MRPL31*, *RSM28* and *MNP1* encode 54S ribosomal protein L31, 37S ribosomal protein *RSM28* and 39S ribosomal protein L12, respectively. These genes participate in the biogenesis of mitochondrial ribosomes and play an active role in mitochondrial translation. The gene *MHR1* is involved in repairing mitochondrial DNA double-strand breaks and encodes mitochondrial homologous recombination protein 1, which is crucial for maintaining mitochondrial function and repairing mitochondrial DNA lesions (Prasai et al. 2018). *PET122* is necessary for the translation of cytochrome *c* oxidase subunit III (Ohmen et al. 1988), a component of the electron transport chain that is essential for ATP production. Under acetic acid stress, these genes associated with mitochondrial ribosomes and translation are suppressed, which may lead to decreased ATP production and disrupted cellular functions, such as reduced mitochondrial respiration and impaired cell growth.

The hub nodes of up-regulated DEGs are mainly related to the cellular component and biological processes of proteasomes (Table S6). The 26S proteasome is a large multi-subunit complex responsible for protein degradation in eukaryotic cells (Bard et al. 2018). Excluding *HAT1* and *DOA4*, the other five hub genes constitute functional module 1 in the major PPI network of up-regulated DEGs (Fig. 5c). The GO and KEGG enrichment analysis results suggest that the GO terms and KEGG pathways related to proteasomes were enriched in module 1 (Tables S8-S9). *DOA4* encodes the deubiquitinating enzyme Doa4p, which is central to the yeast ubiquitin-dependent proteolytic system (Papa and Hochstrasser 1993). This enzyme is associated with the yeast 26S proteasome and removes ubiquitin from protein hydrolysis intermediates on the proteasome before or after substrate degradation to promote protein hydrolysis (Papa et al. 1999). Under acetic acid stress, up-regulated genes such as *RPN6*, *RPN11* and *RPN5*, which are associated with the proteasome, may accelerate the assembly of proteasomes and speed up the degradation of intracellular proteins. The up-regulation of *DOA4* may facilitate the ubiquitin-dependent protein catabolic process and assists the proteasome in further acceleration of protein degradation. Meanwhile, *HAT1* encodes the catalytic subunit of the HAT-B complex, specifically modifying Lys12 of free histone H4, and it plays crucial roles in chromatin structure, transcription activation, DNA repair, gene silencing and cell-cycle progression through histone acetylation (Carrozza et al. 2003; Kurdistani and Grunstein 2003; Rosaleny et al. 2005). The up-regulation of *HAT1* may increase histone acetyltransferase 1 activity, leading to a higher level of histone acetylation. This, in turn, can cause changes in gene expression, activate replication origins earlier, and accelerate the correct repair of DNA damage in yeast to cope with the adverse external environment.

## Materials and methods

### Strain, media and culture conditions

*K. marxianus* DMKU3-1042 (Limtong et al. 2007), which was purchased from NITE Biological Resource Center with the deposit number of NBRC 104275, was used throughout this study. YPD medium (10 g/L yeast extract, 20 g/L peptone and 20 g/L glucose) was used for pre-culture of the yeast. After overnight pre-culture in flasks with shaking at 45°C, yeast cells were washed with sterilized water and inoculated into 100-mL serum bottles with 30 mL fermentation medium (10 g/L yeast extract, 20 g/L peptone, 80 g/L glucose and 40 g/L xylose) in each bottle. The initial optical density at 600 nm (OD_600_) of yeast cells in each bottle was set as ~ 3.0. To investigate the effect of acetic acid on ethanol fermentation, the concentrations of acetic acid in the fermentation media were set as 0%, 0.25% and 0.3% (w/v), respectively. All fermentation experiments were conducted at 45°C with three biological replicates.

### Quantitative analyses of substrates and extracellular metabolites

Broth samples were collected at intervals throughout the fermentation process. The samples were centrifuged at 10,000 × g for 1 min. Then the supernatants were diluted by 0.05 mol/L H_2_SO_4_ for 20 times and filtered through filters with 0.45-µm pores. The cell pellets were flash-frozen in liquid nitrogen and stored at -80℃ for subsequent analysis. The concentrations of glucose, xylose, acetic acid, ethanol and glycerol in the fermentation broth were measured by a HPLC system equipped with an RID-20A refractive index detector (Shimadzu, Japan) and an Aminex HPX-87H column (Bio-Rad, Hercules, CA, USA). The mobile phase was 0.05 mol/L H_2_SO_4_ with a flow rate of 0.6 mL/min. The column temperature and detector temperature were both set as 40°C.

 The fermentation parameters were calculated as follows:

1 $$\mathrm{volumetric}\;\mathrm{productivity}\;(\mathrm g/\mathrm{L\ h}^{-1})=\frac{\mathrm{ethanol\ produced}(\mathrm g/\mathrm L)}{\mathrm{time}(\mathrm h)}$$

2 $$\mathrm{specific}\;\mathrm{productivity}\;(\mathrm g/\mathrm{OD}_{600}\ \mathrm h^{-1})=\frac{\mathrm{volumetric\ productivity}(\mathrm g/\mathrm{L\ h}^{-1})}{\mathrm{final}\ {\mathrm{OD}}_{600}}$$

3 $$\mathrm{conversion}\;\mathrm{yield}\;(\mathrm{g\ ethanol}/\mathrm{g\ sugar})=\frac{\mathrm{ethanol\ produced}(\mathrm g/\mathrm L)}{\mathrm{sugar\ consumed}(\mathrm g/\mathrm L)}$$

### High-throughput RNA sequencing (RNA-seq) and bioinformatic analysis

To investigate the transcriptomic responses of the yeast to acetic acid, yeast cells collected at the 2nd hour of high-temperature fermentation under the condition of 0.25% acetic acid (treatment group) and no acetic acid (control group) were subjected to high-throughput RNA-seq. Three biological replicates were carried on for RNA-seq experiments. Total RNA samples were extracted from the cell pellets using the EZNA Yeast RNA Kit (Omega Bio-tek, Doraville, CA, USA) and then sent to Shanghai Majorbio Bio-pharm Technology Co., Ltd. (Shanghai, China) for quality and quantity evaluation, cDNA library construction and high-throughput sequencing. The genome sequence of *K. marxianus* DMKU3-1042 in the NCBI database (accession number: PRJDA65233) (Lertwattanasakul et al. 2015) was used as the reference genome. After removing the adaptors and the low-quality reads, the clean reads were aligned to the reference genome using HISAT2 (Kim et al. 2015). The differentially expressed genes (DEGs) were identified using DESeq2 (Love et al. 2014). The resulting *P* values were adjusted using the Benjamin and Hochberg’s approach for controlling the false discovery rate. Genes with adjusted *P* (*P*-adjust) values less than 0.05 found by DESeq2 were considered as differentially expressed. Gene Ontology (GO) enrichment analysis of the DEGs was performed using the GOseq R package (Young et al. 2010). KOBAS software was used for Kyoto Encyclopedia of Genes and Genomes (KEGG) pathway enrichment analysis (Mao et al. 2005). GO terms and KEGG pathways with *P-*adjust values less than 0.05 were considered significantly enriched.

### Protein–protein interaction (PPI) network analysis

The STRING database (http://string-db.org) (Szklarczyk et al. 2021) was used to construct PPI networks of the identified DEGs. Given that the PPI information of *K. marxianus* was not included in the STRING database, we chose *Kluyveromyces lactis* as the reference. Then the PPI network data was imported into the Cytoscape software for subsequent analysis (Shannon et al. 2003). The centrality parameters (degree, betweenness, eigenvector) were analyzed using CentiScaPe 2.2 (Scardoni et al. 2009). Nodes with higher centrality values than average were identified as hub nodes. The major PPI networks were constructed based on the intersection of the hub nodes identified based on the three selected centrality parameters. The most significant modules in a major PPI network were identified using Molecular Complex Detection (MCODE) plugin (Bader and Hogue 2003) with a K-score value of 5. The hub genes in a PPI network were ranked based on the MCC algorithm in CytoHubba plugin of Cytoscape (Chin et al. 2014).

### Quantitative protein assay

To quantify the protein content in yeast cells, we first collected yeast cells at the 2nd hour of high-temperature fermentation under the condition of 0.25% acetic acid (treatment group) and no acetic acid (control group), respectively. Then total protein was extracted using the Yeast Total Protein Extraction Kit (Sangon Biotech, Shanghai, China) and quantitative protein assay was performed using Bradford Protein Assay Kit (Sangon Biotech, Shanghai, China) following the manufacturer's instructions.

## Supplementary Information

**Additional file 1**.

## Data Availability

The raw data were deposited to the China National GeneBank database (CNGBdb) under the accession number of CNP0004221.

## Abbreviations

RNA-seq: RNA sequencing
DEG: Differentially expressed gene
GO: Gene ontology
KEGG: Kyoto Encyclopedia of Genes and Genomes
PPI: Protein-protein interaction
